# Supplementary figures and images for: Red blood cell homeostasis in children and adults with and without asymptomatic malaria infection in Burkina Faso
Source: PLoS One. 2020 Nov 30;15(11):e0242507. doi: 10.1371/journal.pone.0242507 (PMC7703889; doi:10.1371/journal.pone.0242507)

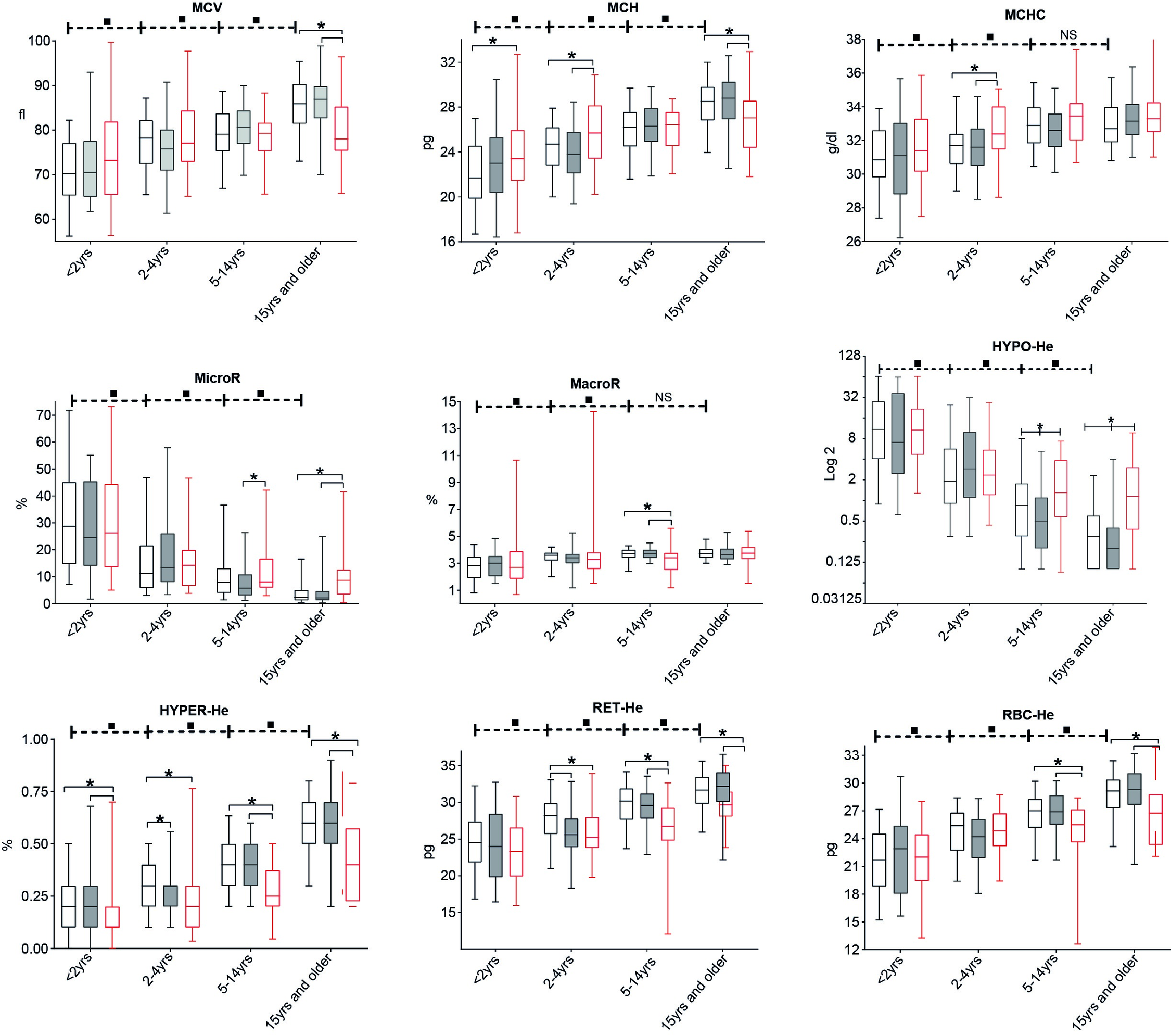

Supplement: S1 Fig — Plots display the status of each haematology parameter per age category. In each age category, participants are divided regarding the health status according to the case definitions whereby healthy smear-negative subjects are represented by “No malaria”, smear-positive asymptomatic cases represented by “Asymptomatic malaria” and smear-positive patienst with fever are represented by “Clinical malaria”. Whiskers bottom and top limits are 5th and 95th percentiles respectively; (): continuous line is used for comparison between clinical status within the same age category and each clinical status was compared with all the other status; (—): dotted line is used for comparison between age category in the “No malaria” group; *: p value with statistically significant difference (p<0.05) between clinical status within the same age category; ▪: p value with statiscally significant difference (p<0.05) between age category in the “No malaria” group; Mann-Whitney U test was used for the comparison between groups; NS: not significant; yrs: years; %: percentage. No malaria Asymptomatic malaria Clinical malaria. (TIF) [file pone.0242507.s001.tif]

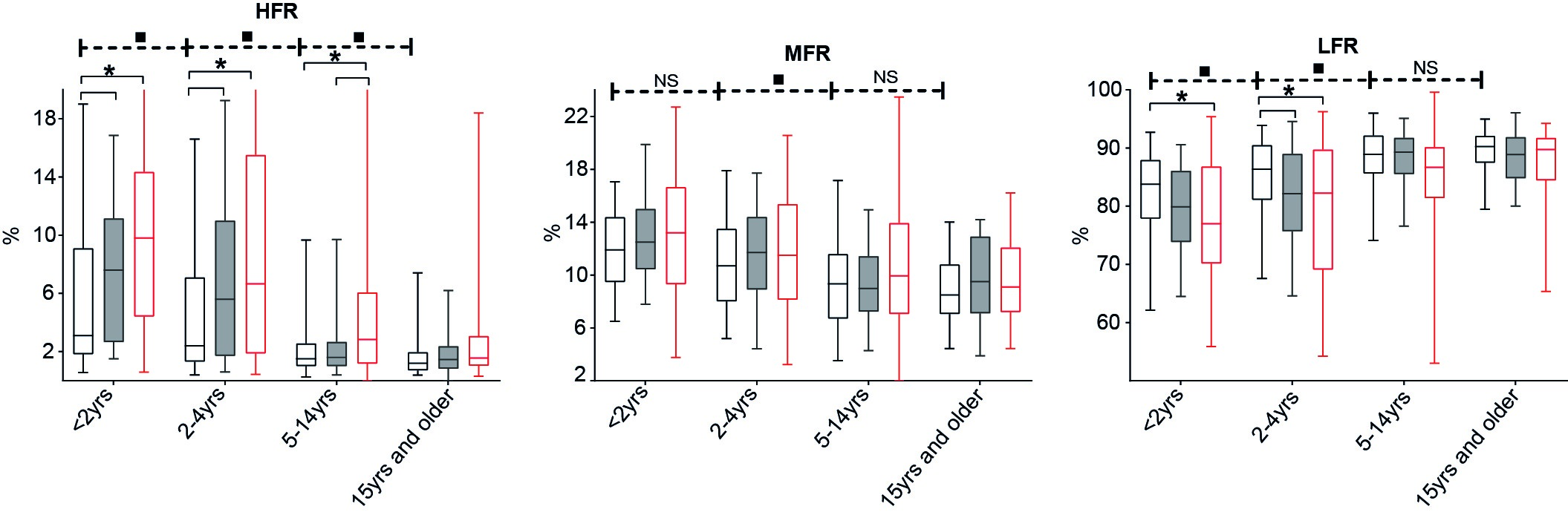

Supplement: S2 Fig — Plots display the status of each haematology parameter per age category. In each age category, participants are divided regarding the health status according to the case definitions whereby healthy smear-negative subjects are represented by “No malaria”, smear-positive asymptomatic cases represented by “Asymptomatic malaria” and smear-positive patienst with fever are represented by “Clinical malaria”. Whiskers bottom and top limits are 5th and 95th percentiles respectively; (): continuous line is used for comparison between clinical status within the same age category; (—): dotted line is used for comparison between age category in the “No malaria” group; *: p value with statistically significant difference (p<0.05) between clinical status within the same age category; ▪: p value with statiscally significant difference (p<0.05) between age category in the “No malaria” group; Mann-Whitney U test was used for the comparison of median between groups; NS: not significant; yrs: years; %: percentage. No malaria Asymptomatic malaria Clinical malaria. (TIF) [file pone.0242507.s002.tif]
