# Supplementary material for: Red blood cell homeostasis in children and adults with and without asymptomatic malaria infection in Burkina Faso
Source: PLoS One. 2020 Nov 30;15(11):e0242507. doi: 10.1371/journal.pone.0242507 (PMC7703889; doi:10.1371/journal.pone.0242507)
Supplement: S1 File — (DOCX) [file pone.0242507.s004.docx]

**SUPPORTING INFORMATION**

**S1 File: Supporting Methods**

**Study design**

In the explorative cross-sectional field study, participants were selected after a complete list of inhabitants per village was created. A total of 1000 participants, with 200 participants in each of the following five age categories: less than 2 years, 2-4 years, 5-9 years, 10-14 years and ≥15 years were randomly selected. In order to prevent bias, a proportionate number of participants were selected from each village based on its total population. There was no significant variation in the socio-economic and demographic distribution between villages. Participants were randomly selected from each sample stratum using systematic selection. Selected participants were informed by a field worker and invited to a central location in their village for an eligibility check. Exclusion criteria were an axillary temperature of <34.5 or > 38.0°C, symptoms of acute disease, current chronic illnesses such as HIV, tuberculosis, renal failure, cardiac disease (if known). Because the RTS’S vaccination trial (phase 3 malaria vaccine candidate) from GlaxoSmith Kline (Rixensaart, Belgium) was previously conducted in this area, a previous participation in this trial was added in the exclusion criteria. This was particularly important because of the potential impact of this vaccine on the immunity to malaria.

After consent was obtained, an electronic questionnaire with detailed demographic and health associated questions was taken and blood pressure, heart rate, axillary temperature, weight, height and mid-upper arm circumference (MUAC) were measured. Finally, a venous blood sample was collected in an EDTA and a heparin anticoagulated tube and processed at the Clinical Research Unit of Nanoro (CRUN) laboratory within 6 hours of collection.

Participants from the cross-sectional study were recruited from June to September which corresponds to the malaria transmission season. Patients from the diagnostic accuracy study were recruited throughout a year, but most were recruited during malaria transmission season.

The studies were carried out in in a hyperendemic area for malaria, with 90-95% *Pf* prevalence and an annual malaria incidence per 1000 inhabitants of around 600 (Rouamba et al BMC Public Health 2019, Febr 19). Mortality rate was 35.9% in 2017 (Tableau de bord 2017 des Indicateurs de Santé du Ministère de la santé du Burkina Faso)

**Laboratory analyses**

Microscopy results were expressed as asexual parasites per microliter using the patients White Blood Cell (WBC) count as measured by the hematology analyser. Slides were examined by two independents qualified microscopists. Presence of one or more *Plasmodium* parasites was considered as a positive test. In case of discrepancies between the two readers results (*e.g.* discrepancy between positive and negative slides, a more than 1 log difference in parasite density, and discrepancy in *Plasmodium* species) a third microscopist’s reading blinded to the results of previous readers was required. The final result was the average of the two closest reader’s results.

The measurement of iron biomarkers was performed by using the Thermo Scientific^TM^ Pierce^TM^ and the R&D systems Quantikine® IVD® human ELISA Kits respectively for Ferritin, hepcidin and soluble transferrin receptor. Circulating cytokines were measured with the Human High Sensitivity Cytokine Premixed Magnetic Luminex Performance Assay kits from Bio-Techne (R&D systems). Ex-vivo cytokines production was measured to assess the immunity and was only done in the healthy volunteers participants (the cross-sectional study).

**Data management and statistical aspects**

For the cross-sectional study, all data were collected in the field on electronic devices by trained nurses using an electronic data collection tool built with open data kit (ODK). Data were uploaded once a week on a secure local server based at CRUN. Hemocytometry data was taken directly from the hematology analysers in an excel file and converted by SYSMEX Company for clinical use. Malaria microscopy data was recorded in duplicate in laboratory registers and entered into a secured excel database (Microsoft, Washington, USA). After patient inclusion was completed, ODK data were downloaded from the server in a password protected excel file. Data were then merged with haematology results and malaria microscopy results, after which it was checked and cleaned.

GraphPad software (GraphPad Prism version 7.00 for Windows, GraphPad Software, La Jolla California USA) was used to generate graphs and figures. The recruitment of participants was stratified by 5 age groups, but after data collection the 5-9 and 10-14 age groups were combined to increase the number of participants that were smear negative.

**Ethical considerations**

Written informed consent was obtained from all participants or their parents/legal guardians. Assent was obtained from all participants aged 7 to 20 years according to the local requirement. Iron supplementation, antipyretic, antibiotic and antimalarial treatment were provided by the study team when indicated.

**SUPPORTING TABLES AND FIGURES**

**S1 Table**: Parameters obtainable from Sysmex XN-1000 analyser

| **Analytical parameters** | |
| --- | --- |
| Parameter name | Parameter description |
| WBC | White blood cell (leukocyte) count |
| RBC | Red blood cell (erythrocyte) count |
| Hb | Haemoglobin concentration |
| HCT | Haematocrit |
| MCV | Mean corpuscular volume |
| MCH | Mean corpuscular haemoglobin |
| MCHC | Mean corpuscular haemoglobin concentration |
| PLT | Platelet count |
| RDW-SD | Red cell distribution width (standard deviation) |
| RDW-CV | Red cell distribution width (coefficient of variation) |
| MicroR | Micro RBC ratio (the proportion of small RBCs as a % of total RBCs) |
| MacroR | Macro RBC ratio (the proportion of large RBCs as a % of total RBCs) |
| PDW | Platelet distribution width |
| MPV | Mean platelet volume |
| P-LCR | Platelet-large cell ratio (the proportion of large platelets as a % of all platelets) |
| PCT | Plateletcrit |
| NRBC# | Nucleated red blood cell count |
| NRBC% | Nucleated red blood cell percent |
| NEUT# | Neutrophil count |
| Lymph# | Lymphocyte count |
| MONO# | Monocyte count |
| EO# | Eosinophil count |
| BASO# | Basophil count |
| NEUT% | Neutrophil percent |
| Lymph% | Lymphocyte percent |
| MONO% | Monocyte percent |
| EO% | Eosinophil percent |
| BASO% | Basophil percent |
| IG# | Immature granulocyte count |
| IG% | Immature granulocyte percentage |
| AS-LYMP# | Count of lymphocytes mainly synthesizing antibodies with high fluorescence intensity |
| AS-LYMP% | Percentage of lymphocytes mainly synthesizing antibodies with high fluorescence intensity |
| RE-LYMP# | Count of lymphocytes reacting to infection with high fluorescence intensity |
| RE-LYMP% | Percentage of lymphocytes reacting to infection with high fluorescence intensity |
| NEUT-RI | Neutrophil reactivity index |
| NEUT-GI | Neutrophil granularity index |
| RET% | Reticulocyte percent |
| RET# | Reticulocyte count |
| IRF | Immature reticulocyte fraction |
| LFR | Low fluorescence ratio (% of total reticulocytes that have low fluorescence intensity) |
| MFR | Medium fluorescence ratio (% of total reticulocytes that have intermediate fluorescence intensity) |
| HFR | High fluorescence ratio (% of total reticulocytes that have high fluorescence intensity) |
| RET-He | Reticulocyte haemoglobin equivalent (optical measurement) |
| RBC-He | Mature RBC haemoglobin equivalent (optical measurement) |
| Delta-He | Parameter calculated by the equation RET-He – RBC-He |
| HYPO-He | The ratio of the count in the low-level area of the forward scattered light signal in the RBC area of the RET scattergram, to mature red blood cells |
| HYPER-He | The ratio of the count in the high-level area of the forward scattered light signal in the RBC area of the RET scattergram, to mature red blood cells |
| IPF | Immature platelet fraction |
| IPF# | Immature platelet fraction count |
|  |  |
| **RESEARCH PARAMETERS available with the standard XN-1000 software** | |
| RPI | Reticulocyte production index |
| HGB-O | Haemoglobin concentration calculated from the RET channel |
| Delta-HGB | Delta-HGB is calculated by the equation Hb-HGB-O |
|  |  |
| **RESEARCH PARAMETERS derived from standard XN-1000 Software and/or with Flowing Software** [**http://flowingsoftware.btk.fi/index.php?page=2**](http://flowingsoftware.btk.fi/index.php?page=2) | |
| RE-MONO | Number of monocytes with a side fluorescent signal >150 channels representing activated monocytes |
| Phago-MONO | Number of activated monocytes that have an increased side scatter signal as a result of phagocytic activity |
| Free-Hb | This is an estimate of free plasma haemoglobin derived from the equation HGB – HGB-O |

**S2 Table:** White blood cells and Platelet indices reference values (median, and 5^th^-95^th^ percentile) per age category in the smear-negative healthy group

| Parameters | Age category | | | |
| --- | --- | --- | --- | --- |
|  | **Less 2 yrs**  **(n=96)** | **2-4 yrs**  **(n=139)** | **5-14 yrs**  **(n=112)** | **15+ yrs and older (n=136)** |
| WBC (10^3^/µl) | 9.7 (6.0-15.8) | 9.1 (6.2-14.6) | 7.3 (4.9-10.7) | 6.0 (3.7-9.1) |
| NEUT# (10^3^/µl) | 2.5 (1.0-5.3) | 2.8 (1.5-5.6) | 2.6 (1.4-5.1) | 2.2 (1.13.9) |
| NEUT% | 26.4 (13.2-45.4) | 31.4 (17.3-49.6) | 35.1 (22.1-56.4) | 37.6 (22.8-54.4) |
| Lymph# (10^3^/µl) | 5.9 (3.5-10.2) | 5.0 (2.9-9.0) | 3.5 (2.2-6.0) | 2.8 (1.6-4.6) |
| Lymph% | 61.1 (42.8-77.7) | 55.7 (38.9-68.9) | 49.5 (34.5-65.8) | 48.1 (31.6-65.1) |
| MONO# (10^3^/µl) | 0.8 (.5-1.4) | 0.8 (0.4-1.5) | 0.6 (0.4-1.0) | 0.5 (0.3-1.0) |
| MONO% | 8.3 (5.2-13.8) | 8.2 (4.9-14.0) | 8.2 (5.0-13.1) | 9.2 (5.6-14.8) |
| RE-MONO  (10^3^/µl) | 0.04 (0.0-0.12) | 0.03 (0.0-0.14) | 0.03 (0.01-0.08) | 0.02 (0.0-0.06) |
| EO# (10^3^/µl) | 0.3 (0.06-0.9) | 0.3 (0.03-0.8) | 0.3 (0.05-1.3) | 0.2 (0.03-0.9) |
| EO% | 3.2 (.7-8.8) | 2.7 (0.3-8.9) | 4.3 (0.8-15.9) | 3.0 (0.5-12.5) |
| BASO# (10^3^/µl) | 0.03 (0.01-.07) | 0.03 (0.01-0.07) | 0.03 (0.02-0.07) | 0.03 (0.01-0.07) |
| BASO% | 0.3 (0.1-0.7) | 0.3 (0.1-0.7) | 0.5 (0.2-0.9) | 0.5 (0.2-1.1) |
| PLT (10^3^/µl) | 402 (130.3-719.2) | 357 (131.0-612.0) | 298 (202.0-476.8) | 219.5 (85.2-344.0) |
| PDW (fL) | 10.7 (8.4-14.1) | 10.6 (8.4-14.5) | 11.5 (9.4-15.3) | 12.1 (9.0-16.4) |
| MPV (fL) | 9.6 (8.6-10.9) | 9.7 (8.5-11.4) | 10.3 (9.1-11.8) | 10.7 (9.0-12.7) |
| PCT (%) | 4.1 (2.6-7.1) | 3.5 (1.8-5.3) | 3.2 (2.1-4.8) | 2.4 (1.3-3.4) |
| IPF# | 7.3 (2.0-19.8) | 7.1 (2.3-21.9) | 8.0 (2.4-24.9) | 8.1 (2.7-21.8) |
| IPF (%) | 2.0 (.6-7.1) | 2.1 (.5-8.0) | 2.5 (.8-11.1) | 3.8 (1.1-17.6) |

**Data are presented as** median, and 5^th^-95^th^ percentiles.

**Yrs:** years; #: absolute count; %: percentage count.

**S3 Table:** Cytokines (pro and anti-inflammatory) reference values (median, and 5^th^-95^th^ percentile) per age category in the smear negative healthy group.

| Parameters (pg/ml) | Age category | | | |
| --- | --- | --- | --- | --- |
|  | **Less 2 yrs** | **2-4 yrs** | **5-14 yrs** | **15+ yrs and older** |
| IL-10 | 3.1 (0.4-23.7) | 3.0 (0.5-23.3) | 3.4 (0.6-26.1) | 2.2 (0.2-18.9) |
| IL-6 | 1.3 (0.4-11.8) | 1.1 (0.4-3.8) | 0.8 (0.4-5.3) | 1.0 (0.4-3.4) |
| TNF-α | 26.8 (12.0-46.6) | 23.9 (13.0-41.4) | 19.4 (9.9-34.5) | 14.9 (5.0-31.5) |

**Data are presented as** median, and 5^th^-95^th^ percentiles.

**Yrs:** years.

IFN-γ levels were mostly non-detectable in the different smear negative groups

**S4 Table**: Factor analysis table of all the biomarkers per health status group

| **Parameters** | **v. test** | **Mean in category** | **Mean overall** | **SD in category** | **Overall SD** | **P value** |
| --- | --- | --- | --- | --- | --- | --- |
| **NO MALARIA** | | | | | | |
| Hb | 23.3 | 11.6 | 10.5 | 1.3 | 2.3 | <0.0001 |
| LFR | 21.6 | 88.8 | 84.9 | 4.8 | 8.9 | <0.0001 |
| RET-He | 18.7 | 29.6 | 27.8 | 4.1 | 5.0 | <0.0001 |
| RBC-He | 18.2 | 26.9 | 25.3 | 3.6 | 4.3 | <0.0001 |
| MCH | 17.1 | 26.6 | 25.5 | 2.5 | 3.4 | <0.0001 |
| MCV | 13.7 | 81.2 | 78.8 | 6.7 | 9.0 | <0.0001 |
| MCHC | 12.9 | 32.8 | 32.3 | 1.4 | 1.9 | <0.0001 |
| RBC | 12.8 | 4.4 | 4.2 | 0.5 | 0.9 | <0.0001 |
| DELTA-He | 7.8 | 2.8 | 2.5 | 1.5 | 2.0 | <0.0001 |
| NRBC% | -2.9 | 0.1 | 0.4 | 0.1 | 6.1 | 0.004 |
| NRBC# | -2.9 | 0.0 | 0.1 | 0.0 | 1.1 | 0.004 |
| IL6 | -5.9 | 7.8 | 65.9 | 32.6 | 493.4 | <0.0001 |
| Ferritin | -8.7 | 78.0 | 133.0 | 168.7 | 316.9 | <0.0001 |
| IL10 | -9.0 | 58.7 | 152.1 | 261.0 | 516.1 | <0.0001 |
| TNFa | -10.1 | 26.0 | 34.9 | 22.3 | 43.6 | <0.0001 |
| RE-MONO | -11.1 | 0.1 | 0.1 | 0.1 | 0.2 | <0.0001 |
| IFNg | -11.5 | 3.1 | 9.6 | 12.4 | 28.4 | <0.0001 |
| RET# | -12.0 | 7.0 | 8.3 | 3.5 | 5.7 | <0.0001 |
| RE-MONOM | -12.1 | 6.6 | 9.0 | 5.1 | 10.1 | <0.0001 |
| RDW-SD | -12.1 | 41.6 | 44.0 | 5.0 | 9.8 | <0.0001 |
| RET% | -13.7 | 16.4 | 22.8 | 9.4 | 23.3 | <0.0001 |
| MFR | -17.3 | 9.0 | 10.5 | 3.2 | 4.1 | <0.0001 |
| HYPO-He | -18.5 | 1.3 | 5.9 | 1.8 | 12.5 | <0.0001 |
| sTfR | -18.8 | 37.7 | 44.1 | 12.3 | 17.1 | <0.0001 |
| IRF | -21.6 | 11.2 | 15.1 | 4.8 | 8.9 | <0.0001 |
| HFR | -22.3 | 2.2 | 4.6 | 2.2 | 5.5 | <0.0001 |
| RDW-CV | -23.6 | 14.2 | 16.0 | 1.7 | 3.7 | <0.0001 |

| **ASYMPTOMATIC MALARIA** | | | | | | |
| --- | --- | --- | --- | --- | --- | --- |
| HYPO-He | 19.0 | 19.7 | 5.9 | 20.4 | 12.5 | <0.0001 |
| sTfR | 17.3 | 61.3 | 44.1 | 16.4 | 17.1 | <0.0001 |
| RDW-CV | 17.2 | 19.7 | 16.0 | 3.4 | 3.7 | <0.0001 |
| IRF | 15.3 | 23.0 | 15.1 | 8.5 | 8.9 | <0.0001 |
| HFR | 15.2 | 9.4 | 4.61 | 6.0 | 5.5 | <0.0001 |
| MFR | 13.1 | 13.6 | 10.5 | 3.4 | 4.1 | <0.0001 |
| RET# | 7.3 | 10.7 | 8.3 | 7.0 | 5.6 | <0.0001 |
| RDW-SD | 3.2 | 45.8 | 44.0 | 9.8 | 9.7 | 0.001 |
| RET% | 3.0 | 26.8 | 22.8 | 20.0 | 23.3 | 0.003 |
| RBC | 2.1 | 4.3 | 4.2 | 0.7 | 0.9 | 0.03 |
| RE-MONOM | -2.04 | 7.8 | 9.0 | 6.4 | 10.1 | 0.04 |
| DELTA-He | -2.2 | 2.2 | 2.5 | 2.4 | 2.0 | 0.03 |
| IFNg | -2.3 | 5.7 | 9.6 | 13.6 | 28.4 | 0.02 |
| IL10 | -3.0 | 62.9 | 152.1 | 153.1 | 516.1 | 0.003 |
| Ferritin | -3.8 | 63.7 | 133.0 | 104.1 | 316.9 | 0.0002 |
| Hepcidin | -5.3 | 8.3 | 16.7 | 10.2 | 26.8 | <0.0001 |
| Hb | -10.7 | 9.1 | 10.5 | 1.5 | 2.3 | <0.0001 |
| MCHC | -14.1 | 30.7 | 32.3 | 2.1 | 1.9 | <0.0001 |
| LFR | -15.3 | 77.0 | 84.9 | 8.5 | 8.9 | <0.0001 |
| RET-He | -16.2 | 23.0 | 27.8 | 4.2 | 5.0 | <0.0001 |
| MCV | -17.4 | 69.8 | 78.8 | 7.5 | 9.0 | <0.0001 |
| RBC-He | -17.8 | 20.8 | 25.3 | 3.5 | 4.3 | <0.0001 |
| MCH | -20.4 | 21.4 | 25.5 | 2.9 | 3.4 | <0.0001 |

| **CLINICAL MALARIA** | | | | | | |
| --- | --- | --- | --- | --- | --- | --- |
| RE-MONOM | 21.2 | 28.2 | 9.0 | 18.5 | 10.1 | <0.0001 |
| IFNg | 20.8 | 62.2 | 9.6 | 58.7 | 28.4 | <0.0001 |
| Ferritin | 18.3 | 651.1 | 133.0 | 691.2 | 316.9 | <0.0001 |
| IL10 | 17.9 | 974.0 | 152.1 | 1170.5 | 516.1 | <0.0001 |
| RET% | 17.0 | 58.2 | 22.7 | 48.8 | 23.3 | <0.0001 |
| TNFa | 16.4 | 98.7 | 34.9 | 100.8 | 43.6 | <0.0001 |
| RE-MONO | 16.3 | 0.4 | 0.1 | 0.5 | 0.1 | <0.0001 |
| RDW-SD | 14.2 | 56.4 | 44.0 | 19.3 | 9.7 | <0.0001 |
| HFR | 13.6 | 11.2 | 4.61 | 7.5 | 5.5 | <0.0001 |
| RDW-CV | 13.1 | 20.3 | 16.0 | 4.9 | 3.7 | <0.0001 |
| IRF | 12.5 | 25.0 | 15.1 | 12.0 | 8.9 | <0.0001 |
| IL6 | 11.4 | 569.1 | 65.8 | 1453.2 | 493.4 | <0.0001 |
| Hepcidin | 10.0 | 40.6 | 16.6 | 60.2 | 26.8 | <0.0001 |
| MFR | 9.1 | 13.8 | 10.5 | 5.4 | 4.1 | <0.0001 |
| RET# | 8.6 | 12.7 | 8.3 | 9.5 | 5.7 | <0.0001 |
| sTfR | 5.6 | 52.7 | 44.1 | 18.5 | 17.1 | <0.0001 |
| NRBC# | 5.5 | 0.6 | 0.1 | 3.5 | 1.1 | <0.0001 |
| NRBC% | 5.4 | 3.3 | 0.4 | 18.3 | 6.1 | <0.0001 |
| HYPO-He | 2.7 | 8.8 | 5.9 | 10.3 | 12.5 | 0.007 |
| MCV | 2.5 | 80.8 | 78.8 | 12.5 | 9.0 | 0.013 |
| RBC-He | -3.8 | 23.8 | 25.3 | 3.5 | 4.3 | 0.0001 |
| RET-He | -6.8 | 24.7 | 27.7 | 4.1 | 5.0 | <0.0001 |
| DELTA-He | -9.0 | 0.9 | 2.5 | 2.8 | 2.0 | <0.0001 |
| LFR | -12.5 | 75.0 | 84.9 | 12.0 | 8.9 | <0.0001 |
| HGB | -21.4 | 6.1 | 10.5 | 2.1 | 2.3 | <0.0001 |
| RBC | -22.5 | 2.3 | 4.2 | 1.0 | 0.9 | <0.0001 |
