# Supplementary material for: Red blood cell homeostasis in children and adults with and without asymptomatic malaria infection in Burkina Faso
Source: PLoS One. 2020 Nov 30;15(11):e0242507. doi: 10.1371/journal.pone.0242507 (PMC7703889; doi:10.1371/journal.pone.0242507)
Supplement: Attachment — Submitted filename: Comments_PONE-D-20-20217.docx [file pone.0242507.s006.docx]

Red blood cell homeostasis in children and adults with and without asymptomatic malaria

infection in Burkina Faso.

Kaboré B. et al.

This paper describes a large set of hematological parameters in a population living in a malaria hyperendemic area. The novelty of the study lies fundamentally in the integration of reticulocyte and bone marrow related data in symptomatic and asymptomatic subpopulations. The hypothesis is that in asymptomatic malaria immune tolerance may have a protective effect on erythropoiesis, which would compensate the anaemia derived from the pro-inflamatory status and hemolysis. The results support a correlation between the bone marrow response, with higher turnover of RBC, and the development of asymptomatic malaria. Differences were found mostly between children showing clinical malaria and asymptomatic or no malaria subjects.

Overall the study appears well designed, the analytical techniques correctly used and the results convincing, however there are some weaknesses which I recommend to address before publication.

Specific comments:

1. Although the statistical analysis performed yields significant differences among the three main groups analyzed (smear-negative, asymptomatic and clinical malaria) for several parameters, some data display a rather large dispersion (figures 2, 3, 5, 6..), particularly in the adult groups in clinical malaria and some asymptomatic malaria sets. This might be explained if these groups included some subjects displaying characteristics not contemplated in the selection criteria, as a concomitant non malaria health issue, or perhaps the inclusion of pregnant women, which has been reported to display differential response to malaria, leading to heterogeneity in the group. The authors may review the characteristics of the individuals included in each study group and test if the statistical dispersion is reduced by the exclusion or regrouping by these criteria.

2. Participants of the asymptomatic group: the subjects were included in this group based on being smear-positive and healthy at the moment of the test. It is stated also (Supporting methods, study design) that a questionnaire was taken at sample collection, but no information is given if the subjects were questioned for febrile episodes in the previous two weeks to ascertain lack of malaria symptoms. It is possible that some of the asymptomatic subjects were actually either developing symptomatic malaria at an early stage or tested after a malaria episode. Related to the comment #1, this would misrepresent both the symptomatic and asymptomatic groups and add uncertainty to the statistics. Ideally the asymptomatic group should correspond with immune-tolerant individuals. If the authors have such information it would be advisable to be mentioned in the text.

3. The group of subjects ≥15 years is quite broad, it would be convenient to indicate the average age of this group as blood parameters are most likely affected in what is considered elderly people in endemic areas (>40 years) than in young adults (15-40 years)

Minor comments

“NS” in table 1 and figures S1 and S2 is not defined in the text or legends

Quality of figures: axis labels are difficult to read at the resolution provided in the figures containing large number of plots (fig 2, 5, 7, S1)
