# Supplementary material for: Red blood cell homeostasis in children and adults with and without asymptomatic malaria infection in Burkina Faso
Source: PLoS One. 2020 Nov 30;15(11):e0242507. doi: 10.1371/journal.pone.0242507 (PMC7703889; doi:10.1371/journal.pone.0242507)
Supplement: Attachment — Submitted filename: Response to Reviewers.docx [file pone.0242507.s007.docx]

Rebuttal letter related to paper: Red blood cell homeostasis in children and adults with and without asymptomatic malaria infection in Burkina Faso, by Kaboré B. et al.

Comments academic editor and reviewers in black, our responses are presented in blue. Changes in the main text are presented with track changes

**Comments academic editor:**

This paper describes a large set of hematological parameters in a population living in a malaria hyperendemic area. The novelty of the study lies fundamentally in the integration of reticulocyte and bone marrow related data in symptomatic and asymptomatic subpopulations. The hypothesis is that in asymptomatic malaria immune tolerance may have a protective effect on erythropoiesis, which would compensate the anaemia derived from the pro-inflammatory status and hemolysis. The results support a correlation between the bone marrow response, with higher turnover of RBC, and the development of asymptomatic malaria. Differences were found mostly between children showing clinical malaria and asymptomatic or no malaria subjects.

Overall the study appears well designed, the analytical techniques correctly used and the results convincing, however there are some weaknesses which I recommend to address before publication.

Specific comments:

1. Although the statistical analysis performed yields significant differences among the three main groups analyzed (smear-negative, asymptomatic and clinical malaria) for several parameters, some data display a rather large dispersion (figures 2, 3, 5, 6..), particularly in the adult groups in clinical malaria and some asymptomatic malaria sets. This might be explained if these groups included some subjects displaying characteristics not contemplated in the selection criteria, as a concomitant non malaria health issue, or perhaps the inclusion of pregnant women, which has been reported to display differential response to malaria, leading to heterogeneity in the group. The authors may review the characteristics of the individuals included in each study group and test if the statistical dispersion is reduced by the exclusion or regrouping by these criteria.

**Answer Kaboré B. et al.: Participants from the clinical malaria group were selected from a prospective diagnostic accuracy study (see method section): Included were malaria confirmed cases in whom no other concurrent pathology was present, neither by clinical investigations, nor by additional investigations. Women were recruited from the medical department, not from the maternity ward. Pregnant women were not included even tough not mentioned in the exclusion criteria.**

**For the asymptomatic malaria group: Apparently healthy subjects were selected from the community during a cross sectional study whereby clinical information and blood samples have been collected. Participants were excluded if fever was present or if there was any sign, symptom or history of acute or chronic diseases. Women were also questioned for possible preganancy: if pregnancy was confirmed women were not included in the study. A pregnancy test was not done to further rule out pregnancy however. In conclusion: concomitant pathology can unfortunately not be determined in our population and the suggested additional statistical test can therefore also not be done. We have mentioned this as a limitation in the discussion.**

2. Participants of the asymptomatic group: the subjects were included in this group based on being smear-positive and healthy at the moment of the test. It is stated also (Supporting methods, study design) that a questionnaire was taken at sample collection, but no information is given if the subjects were questioned for febrile episodes in the previous two weeks to ascertain lack of malaria symptoms. It is possible that some of the asymptomatic subjects were actually either developing symptomatic malaria at an early stage or tested after a malaria episode. Related to the comment #1, this would misrepresent both the symptomatic and asymptomatic groups and add uncertainty to the statistics. Ideally the asymptomatic group should correspond with immune-tolerant individuals. If the authors have such information it would be advisable to be mentioned in the text.

**Answer Kaboré B. et al.: We specifically asked all participant if any febrile illness (fever, headaches…) had been present the past 2 days before the sample collection. This information is added in the Supplement (Supporting file: “study design” section). Follow-up data are unfortunately not available as we performed a cross-sectional study.**

3. The group of subjects ≥15 years is quite broad, it would be convenient to indicate the average age of this group as blood parameters are most likely affected in what is considered elderly people in endemic areas (>40 years) than in young adults (15-40 years)

**Answer Kaboré B. et al.: The information was added in the text. See Page 8 Lines 164-166**

Minor comments

“NS” in table 1 and figures S1 and S2 is not defined in the text or legends

**Answer Kaboré B. et al.: The information was added in the appropriate legends**

Quality of figures: axis labels are difficult to read at the resolution provided in the figures containing large number of plots (fig 2, 5, 7, S1)

**Answer Kaboré B. et al.: We increased the resolution in the Figures. Note: the quality of the figures in “tiff format” is better in comparison to the one provided in the generated PDF file.). In addition, all the figures have been formatted using PACE.**

------------------------------------------------------------------------------------------------------------------------------

**Comments reviewer #1**:

1) In Table 1, the percentage of each age group should reflect the proportion of each column instead of each row.
**Answer Kaboré B. et al.: Correction was done in the Table 1**

2) There are many biomarkers examined here. It will be great to do some in-depth analysis on the correlations of the biomarkers to cluster and group biomarkers, and to draw some results and conclusion at the hyper level.

**Answer Kaboré B. et al.: In the attachment you will find 2 files: A PCA Plot (PDF file named “Supporting file Response to reviewers_Cluster analysis Plot”) and the tables (word file named “Supporting file Response to reviewers_Cluster analysis Tables”)**

**In the plot as well as in the tables:**

**Cluster 1🡪corresponds to No malaria**

**Cluster 2🡪corresponds to Asymptomatic malaria**

**Cluster 3🡪corresponds to Clinical malaria**

**The tables represent the details of the parameters used for the clustering analyses in the different clusters. In the “v.test” column, the values in front of each parameter represent the importance of this parameter in the corresponding cluster and are ordered from the highest to the lowest. The positive value (values with no sign) corresponds to an increase of the parameter in the cluster and the negative values correspond to a decrease.**

**As you will see in these tables, the asymptomatic malaria group is less pro-inflammatory and has less decrease of Hb compared to clinical malaria even tough reticulocytes counts are increase in both groups. The overall parameters are in line with our conclusion as made in the paper.**

**Reviewer #2**: Red blood cell homeostasis in children and adults with and without asymptomatic malaria infection in Burkina Faso. Kaboré B. et al.
This paper describes a large set of hematological parameters in a population living in a malaria hyperendemic area. The novelty of the study lies fundamentally in the integration of reticulocyte and bone marrow related data in symptomatic and asymptomatic subpopulations. The hypothesis is that in asymptomatic malaria immune tolerance may have a protective effect on erythropoiesis, which would compensate the anaemia derived from the pro-inflamatory status and hemolysis. The results support a correlation between the bone marrow response, with higher turnover of RBC, and the development of asymptomatic malaria. Differences were found mostly between children showing clinical malaria and asymptomatic or no malaria subjects.
Overall the study appears well designed, the analytical techniques correctly used and the results convincing, however there are some weaknesses which I recommend to address before publication.

Specific comments:

1. Although the statistical analysis performed yields significant differences among the three main groups analyzed (smear-negative, asymptomatic and clinical malaria) for several parameters, some data display a rather large dispersion (figures 2, 3, 5, 6..), particularly in the adult groups in clinical malaria and some asymptomatic malaria sets. This might be explained if these groups included some subjects displaying characteristics not contemplated in the selection criteria, as a concomitant non malaria health issue, or perhaps the inclusion of pregnant women, which has been reported to display differential response to malaria, leading to heterogeneity in the group. The authors may review the characteristics of the individuals included in each study group and test if the statistical dispersion is reduced by the exclusion or regrouping by these criteria.

**Answer Kaboré B. et al.: see above**

2. Participants of the asymptomatic group: the subjects were included in this group based on being smear-positive and healthy at the moment of the test. It is stated also (Supporting methods, study design) that a questionnaire was taken at sample collection, but no information is given if the subjects were questioned for febrile episodes in the previous two weeks to ascertain lack of malaria symptoms. It is possible that some of the asymptomatic subjects were actually either developing symptomatic malaria at an early stage or tested after a malaria episode. Related to the comment #1, this would misrepresent both the symptomatic and asymptomatic groups and add uncertainty to the statistics. Ideally the asymptomatic group should correspond with immune-tolerant individuals. If the authors have such information it would be advisable to be mentioned in the text.

**Answer Kaboré B. et al.: see above**

3. The group of subjects ≥15 years is quite broad, it would be convenient to indicate the average age of this group as blood parameters are most likely affected in what is considered elderly people in endemic areas (>40 years) than in young adults (15-40 years)

**Answer Kaboré B. et al.: see above**

Minor comments
“NS” in table 1 and figures S1 and S2 is not defined in the text or legends
Quality of figures: axis labels are difficult to read at the resolution provided in the figures containing large number of plots (fig 2, 5, 7, S1)

**Answer Kaboré B. et al.: see above**

**Reviewer #3**: This manuscript by Kaboré et al describes the hematological alterations shown in asymptomatic or clinical malaria and also investigates the bone marrow response to the loss of RBCs depending on the severity of the infection. The study integrates a wide variety of hematological variables, iron biomarkers and cytokines as a comprehensive approach to understanding how the bone marrow compensates for the condition of anaemia. One of the major highlights of the paper is the demonstration that asymptomatic malaria is associated with enhanced erythropoiesis and immune tolerance compared to those with clinical malaria, probably due to a lower imbalance towards the production of pro-inflammatory factors in individuals with asymptomatic malaria.
In recent years, asymptomatic malaria carriers have become a focus of malaria research, as they are considered the silent reservoir of malaria. The factors that protect these individuals from developing clinical symptoms are still unclear, however, this study provides new insights into the underlying processes of subclinical malaria. In addition, the large and significant number of samples analysed from different ages provides solid and consistent results.
It should be pointed out that the individual data underlying the findings are not fully available in the manuscript and no repository have been provided. Although summary statistics are available, the data points behind means or ranges are not given as stated in the PLOS Data policy.

**Answer Kaboré B. et al.: Data will be made available.**

Most of graphs and statements are clear and well-founded. Nevertheless, there are some points that the authors may review to facilitate the readability and interpretation of the results:

Major comments:

1. As the authors note in the discussion, despite the study is focussed on asymptomatic malaria, the diagnostic method used fails to detect those individuals with submicroscopic malaria, known to be the majority of asymptomatic malaria individuals. In addition, there is no information about other pathologies that may cause hematological alterations such as sickle cell disease. For that reason, smear-negative samples should not be considered reference values, as they may include many submicroscopic malaria and hemoglobin S carriers.

**Answer Kaboré B. et al.: Microscopy indeed fails to detect submicroscopic parasitemia: this has been mentioned as a limitation in the discussion. In addition, we did not check for other causes of hematological alterations, such as sickle cell anemia. Also this information has been mentioned as a limitation of our study in the discussion: Page 21 Lines 402-407**

2. Some of the statements do not fit exactly with the results shown in the graphs or are too generalistics. Along the results or discussion the authors describe significant differences between infection groups, however they do not indicate the age groups in which this occurs (e.g. line 270, Fig2, one of the under 15 years groups do not show increased reticulocyte numbers; line 272, Fig2, the authors should indicate that Delta-he levels are low only in children; line 274, Fig2, RPI is only elevated in under-2 children; Line 378, Fig2, the authors claim that erythrocyte indices increase with age, however, the RBC numbers are constant along the age groups). For the benefit of the readers, the authors should be more accurate in their description of the results.

**Answer Kaboré B. et al.: The different suggestions were corrected accordingly. See the corresponding Lines**. **The indices stated there do not referred to RBC/erythrocyte numbers but to the others parameters. Please see the paragraph “Red blood cell reference values and iron inflammation biomarkers” under results section on Page 12 Lines 184-191**

3. Lines 403-405: The authors claim that asymptomatic malaria infections, unlike clinical malaria, have an adequate bone marrow response to decreasing Hb levels, using reticulocyte numbers and RPI as indicators, however, reticulocyte numbers show increased numbers in both subclinical and clinical malaria. It is unclear on what differential results between both groups this statement is based.

**Answer Kaboré B. et al.: Reticulocyte numbers may increase when anaemia develops, however the increment of reticulocyte numbers may be insufficient to maintain an adequate Hb level. RPI reflects the reticulocytes production capacity according to the level of anemia. Our data indicate that in asymptomatic malaria reticulocyte numbers and RPI levels are able to compensate for Hb loss, but not in clinical malaria.**

4. This reviewer does not see a clear reason why the cytokine production after LPS stimulation is not analysed in clinical malaria patients. In line with the rest of data provided where this group is considered. This analysis is important to provide insights into subclinical malaria immune tolerance in comparison to those who develop clinical symptoms.

**Answer Kaboré B. et al.: We agree that the data on the cytokine production after LPS stimulation in the clinical malaria patients would have been interesting but unfortunately we did not have enough sample to performed this experiment.**

5. Figures 2, 3, 5, 6 and S1: When the p-value is marked as a line divided into two segments (e.g. Fig2, RBC, <2yrs) it is not clear whether there are significant differences between smear-negative samples and subclinical malaria and between subclinical and clinical malaria or whether there are also significant differences between smear-negative and clinical malaria. Based on my interpretation, I understand that there are no significant differences between smear-negative and clinical malaria, however, at first glance it seems that there are. Could the authors clarify this point? If there are significant differences between these groups, a clear definition in the legends and graphs on how the p-values are shown, should be provided.

**Answer Kaboré B. et al.: Indeed, comparison have been done between all the different groups. Text has been added in the legend section to clarify this issue**

Minor comments:

6. Line 60, 404 and 460: What does “adequate” means? The authors should be more concise describing a main conclusion.

**Answer Kaboré B. et al.: We agree with comment reviewer and adjusted the text as follows: The bone marrow response can compensate the increased red blood cell loss in asymptomatic patients, unlike in clinical malaria. Hopefully this is a better statement.**

7. Some references are missing along the manuscript (e.g. line 88, the authors should provide the reference of the Western population data; line 367, the authors may provide the reference of IL-6 role in iron homeostasis; line 417, the authors should provide the reference describing the significant role of hemolysis in asymptomatic malaria).

**Answer Kaboré B. et al.: We fully agree with comments reviewer and added references: Reference for western population was added on Page 5 line 89. Reference added for the role of IL-6 in iron homeostasis (see Page 20 line 378). Finally, a reference was added describing the significant role of hemolysis for asymptomatic malaria (see Page 22 line 417)**

8. Line 264: Could de authors indicate if the difference in Hb level was calculated with the mean of the groups?

**Answer Kaboré B. et al.: The data were presented as median values. See indication added on page 16 line 271**

9. Line 281: To facilitate a better understanding of this section, I would recommend including the acronyms of the mature and immature reticulocytes that are shown in Fig S2.

**Answer Kaboré B. et al.: Suggestion of reviewer was added on Page 17 lines 289-290**

10. Lines 330-331: The authors should clarify what the value in brackets means.

**Answer Kaboré B. et al.: A further clarification, as suggested by reviewer, was added on Page 19 line 339**
