# Supplementary material for: Red blood cell homeostasis in children and adults with and without asymptomatic malaria infection in Burkina Faso
Source: PLoS One. 2020 Nov 30;15(11):e0242507. doi: 10.1371/journal.pone.0242507 (PMC7703889; doi:10.1371/journal.pone.0242507)
Supplement: Attachment — Submitted filename: Response to Revierwers.docx [file pone.0242507.s008.docx]

Rebuttal letter related to paper: Red blood cell homeostasis in children and adults with and without asymptomatic malaria infection in Burkina Faso, by Kaboré B. et al.

Comments reviewer are presented in black, our responses are presented in blue. Changes in the main text are presented with track changes

**Comments reviewer #1**

Reviewer #1: 1) My question of “in-depth analysis on the correlations of the biomarkers to cluster and group biomarkers, and to draw some results and conclusion at the hyper level” on last version meant to make the manuscript more informative to all the audience instead of just to me, a reviewer. Therefore, the relevant analysis results and interpretation should be presented in the manuscript, even if only in supplementary materials, rather than just in the supporting file to the reviewers.

**We thank reviewer for suggesion and we adapted our paper accordingly. See materials and method section: page 8; Lines 148-152 and results section: page 20-21; Lines 387-401. The corresponding figure and table are presented in the supporting materials.**
